# Oral nonabsorbable antibiotics for prevention of recurrent cholangitis; a brief report study

**DOI:** 10.1007/s15010-025-02491-2

**Published:** 2025-02-20

**Authors:** Jesús Fortún, Miguel Angel Rodríguez-Gandía, Vicente Pintado, Pilar Martín-Dávila, Miguel García-González, Javier Graus, Rosa Martín-Mateos, Javier Saez de la Fuente, Alfonso Muriel, Santiago Moreno

**Affiliations:** 1 Department of Infectious Diseases, Hospital Universitario “Ramon y Cajal”, Avda Colmenar Km 9,1, Madrid, 28034 Spain; 2 Instituto de Investigación Sanitaria Hospital “Ramón y Cajal” (IRYCIS), Madrid, 28034 Spain; 3 Department of Medicine, Universidad Alcalá, Madrid, 28801 Spain; 4 Centro de Investigación Biomédica en Red de Enfermedades Infecciosas (CIBERINFEC), Instituto de 5 Salud Carlos III, Madrid, 28029 Spain; 5 Centro de Investigación Biomédica en Red de Enfermedades Hepáticas y Digestivas (CIBEREHD), Instituto Salud Carlos III, Madrid, Spain; 6 Centro de Investigación Biomédica en Red Epidemiología y Salud Pública (CIBERESP), Instituto Salud Carlos III, Madrid, Spain; 7 Department of Gastroenterology and Hepatology, Hospital Universitario “Ramon y Cajal”, Madrid, 28034 Spain; 8 Biostatistics Unit, Hospital Universitario Ramón y Cajal, Madrid, 28034 Spain; 9 Department of Pharmacy, Hospital Universitario “Ramon y Cajal”, Madrid, 28034 Spain

**Keywords:** Recurrent cholangitis, Selective decontamination of the digestive tract, Oral nonabsorbable antibiotics, Prevention

## Abstract

**Background:**

Patients with recurrent cholangitis are at risk of developing life-threatening sepsis. Selective decontamination of the digestive tract (SDD) involving oral nonabsorbable antibiotics has been primarily applied to children undergoing Kasai portoenterostomy surgery.

**Methods:**

In this study, SDD containing colistin, tobramycin, and nystatin was administered to eight patients with recurrent cholangitis, and the incidence density before and after SDD administration was analyzed.

**Results:**

The overall incidence density of cholangitis requiring hospital admission was 0.37 per 100 patient days during the SDD period and was significantly lower than observed before SDD administration (1.05 per 100 patient days) [RR: 0.35 (95% CI: 0.21–0.59); p: <0.001, two-sided]. This was not associated with an increased risk of resistance during SDD administration.

**Conclusion:**

In this study SDD reduced by 65% the frequency and severity of recurrent cholangitis. In addition, this procedure is patient-friendly and microbiologically safe.

## Introduction

Recurrent cholangitis can be a major problem for patients with a compromised biliary tree, involving such conditions as hepaticojejunostomy, stenosis, sphincteroplasty, or endoprosthesis [1]. Many of these patients receive prolonged antibiotic prophylaxis, usually with absorbable antibiotics, such as cephalosporins, trimethoprim/sufamethoxazole (TMP/SMZ) or quinolones. However, these strategies have a limited efficacy over time either due to the selection of resistant bacteria or due to intolerance or toxicity [1, 2].

Selective decontamination of the digestive tract (SDD) is a preventive infection control strategy that usually comprises the administration of oral nonabsorbable antimicrobial and topical agents to the oropharynx and upper gastrointestinal tract, with or without the administration of a short-term course of broad-spectrum intravenous antibiotics. Its use is mainly focused on the prevention of ventilator-associated pneumonia, where different studies have shown contradictory results [3–5]. Although SDD is now routinely used in ICUs throughout Europe, its application has not become generalized in clinical practice despite the evidence of its efficacy and safety [5]. A recent meta-analysis of 32 randomized clinical trials and 24,389 patients estimated a reduction in hospital mortality between 1% and 18% in patients with SDD [4]; however, beneficial effects were only obtained when pooling studies in which SDD included the intravenous component.

The use of SDD in the prevention of recurrent cholangitis is very limited and applied primarily to children with biliary atresia undergoing Kasai portoenterostomy surgery [1, 2, 6, 7]. A small randomized study in children having undergone Kasai surgery confirmed that the benefit of receiving oral neomycin prophylaxis was greater than oral TMP/SMZ, and both practices showed improvement over placebos [6]. Other case series confirmed the efficacy of neomycin in patients with recurrent cholangitis following successful portoenterostomies, despite the use of a variety of antibiotics [7].

The prophylactic effects of antibiotics may occur via two principal pathways: (a) adequate concentrations of antibiotics are excreted from blood into the cholangioles and (b) the bacterial concentration is diminished within the bilio-enteric conduit. TMP/SMZ is concentrated in the bile and has been shown to lower the absorption of bacteria in the bile, suggesting that this antibiotic combination may be useful for the management of cholangitis [6]. The use of nonabsorbable oral antibiotics, such as colistin, tobramycin or nystatin that reach high concentrations at the duodenal level and in bile in patients with the sphincter of Oddi dysfunction, can significantly reduce bacterial growth at this level. Furthermore, they can prevent the risk of ascending cholangitis in the same way that they reduce bacterial colonization in the upper intestinal tract and lessen the risk of aspiration pneumonia in patients undergoing mechanical ventilation.

## Methods

At its meeting on October 1, 2015, the Hospital Pharmacy Committee of Ramón y Cajal Hospital in Madrid approved the off-label use of SDD for patients with recurrent cholangitis. The committee then requested the Center’s Investigational Review Board (IRB) to authorize this application, and the IRB subsequently approved the study. SDD is a solution, containings per 10 ml vial: colistin 130 mg, tobramycin 156 mg and nystatin 2.6 M.I.U. Its routine use in the center, together with pharyngeal paste containing the same elements, is for the prevention of ventilator-associated pneumonia in ICU patients. Preliminary favorable results in the prevention of cholangitis in patients with Kasai porto-enterostomy justified its use by the Commission [1, 2, 6–8].

The diagnosis of cholangitis was established based on a combination of clinical, laboratory, and imaging criteria. The criteria included:

### Clinical findings

- **Charcot’s Triad**: Presence of fever (with or without chills), right upper quadrant abdominal pain, and jaundice.
- When available, evidence of systemic inflammation or signs of sepsis was also considered.

### Laboratory findings

- **Elevated Bilirubin Levels**: Indicating impaired bile excretion and cholestasis.
- **Elevated Gamma-Glutamyl Transpeptidase (GGT)**: Suggesting biliary epithelial injury or obstruction.
- **Elevated Alkaline Phosphatase (ALP)**: Consistent with a cholestatic pattern of liver injury.

### Imaging findings

- Evidence of biliary duct dilatation and/or obstruction on ultrasound, computed tomography (CT), magnetic resonance or cholangiopancreatography.

This indication request was for recurrent cholangitis of any cause, especially in non-oncological cholangiopathies. Recurrent cholangitis was diagnosed following a new episode of cholangitis in a patient with previous episodes and a predisposing cholangiopathy. Oral dose of SDD (a solution vial of 10 ml) was administered for every 8 h. No paste to the mouth was applied. The vials were delivered by the pharmacy service to patients once a month and refrigerated at 4ºC.

The patients included in the study were adults, with a history of recurrent cholangitis who started treatment with SDD. Only episodes of cholangitis requiring hospital admission, with or without the presence of bacteremia and requiring antibiotics intravenously for treatment were considered for the analysis. Period 1 included episodes of cholangitis from the first documented episode requiring hospital admission to the onset of SDD. Period 2 included episodes of cholangitis after SDD onset until the present, death, or loss to follow-up.

Number or episodes of cholangitis, time to a new episode and antimicrobial therapy received were the principal objectives of the study. Other objectives analyzed included: clinical presentation during cholangitis episodes (including bacteremia, the etiological agent and its susceptibility profile), septic shock, duration of hospitalization, and mortality both during the episode and overall.

Summary statistics of demographic variables are presented using absolute frequency, mean, and standard deviations or median. The incidence density of cholangitis episodes and the antibiotic therapy required to treat the episodes were calculated for both periods as the number of events divided by the total follow-up time in days. The effect was estimated as relative risk calculated as the ratio of the incidence rates for the two periods with a 95% confidence interval. All tests were conducted two-tailed. The analysis was performed using Stata 18.0.

## Results

Table 1 lists the main characteristics of the patients. From October 2015 until the completion of this study, eight patients who received SDD were the target of the present study. All had known cholangiopathy, five had stenotic bile ducts, three had undergone papillotomy and three had biliary prostheses. Most had benign bile duct pathology; only one patient (#8) had undergone hepatic metastasectomy secondary to colon cancer and had a Y-Roux bile-digestive anastomosis, but there was no infiltration of the bile duct, although there was mild stenosis. It is noteworthy that three of the eight patients were liver transplant recipients, who developed post-transplant sclerosing or ischemic cholangiopathy. Patient #8, received adjuvant chemotherapy (folinic acid, fluorouracil and oxaliplatin: FOLFOX, and Bevazizumab). All four were immunosuppressed. Finally, there were three patients who required cholecystectomy, two of whom received papillotomy for biliary lithiasis and only one of these three had biliary stenosis. The remaining patients all had bile duct stenosis and three of them had biliary prostheses.

**Table 1:** Characteristics of patients

	pat #1	pat #2	pat #3	pat #4	pat #5	pat #6	pat#7	pat#8
Gender, age	M, 86	M, 84	M, 69	M, 38	M, 40	F, 56	M, 74	M, 50
Cholangiopathy	lithiasic CCT	lithiasic CCT	post transplant bile duct ischemia	post transplant sclerosing cholangitis	post transplant sclerosing cholangitis	postinfectious cholangitis	lithiasic CCT	Whipple, Y-roux#
Immunosuppressive therapy	No	No	Yes, liver Tx	Yes, liver Tx	Yes, liver Tx	No	No	Yes, chemotherapy
Papillotomy	Yes	Yes	No	No	No	Yes	No	No
Biliary stenosis, biliary prosthesis	No, no	No, no	Yes, yes	Yes, no	Yes, no	Yes, yes	Yes, Yes	No, No
SDD start date	8/10/2015	30/6/2016	21/6/2022	1/10/2022	24/3/2022	18/11/2022	12/04/2023	1/03/2023
**Period 1 (before starting SDD)**								
nº cholangitis (income required)	7	6	4	3	4	6	3	3
Days from 1st cholangitis to SDD	665	820	101	670	300	290	492	77
Incidence density per 100 pds	1.05	0.73	3.96	0.45	1.33	2.07	0.60	3.89
nº of secondary bacteremias	3	1	2	0	0	1	1	1
Days antibiotic during admissions	-	73	75	21	35	57	84	24
Incidence density Ab per 100 pds	-	8.90	74.26	3.13	11.66	19.65	17,1	31,1
TMP-SMZ prophylaxis	Yes	Yes	No	No	Yes	No	No	No
**Period 2 (after starting SDD)**								
nº cholangitis (income required)	13	8	1	1	4	0	2	1
Days from SDD to end follow-up	3,070	2,825	240	520	545	120	360	405
Incidence density per 100 pds	0.42	0.28	0.42	0.19	0.73	0	0.55	0.25
nº of secondary bacteremias	9	1	0	0	0	0	0	0
Days antibiotic during admissions	-	51	15	14	65	0	15	6
Incidence density Ab per 100 pds	-	1.80	6.25	2.69	11.92	0	4.16	1.48
TMP-SMZ prophylaxis	Yes	Yes	No	No	Yes	No	No	No

SDD: Selective decontamination of the digestive tract CCT: cholecystectomy pd: person-days Ab: antibiotics

Tx: Transplant #Colonic adenocarcinoma, hepatic metastatectomy and bilio-digestive anastomosis (Y-Roux)

**Fig. 1:**
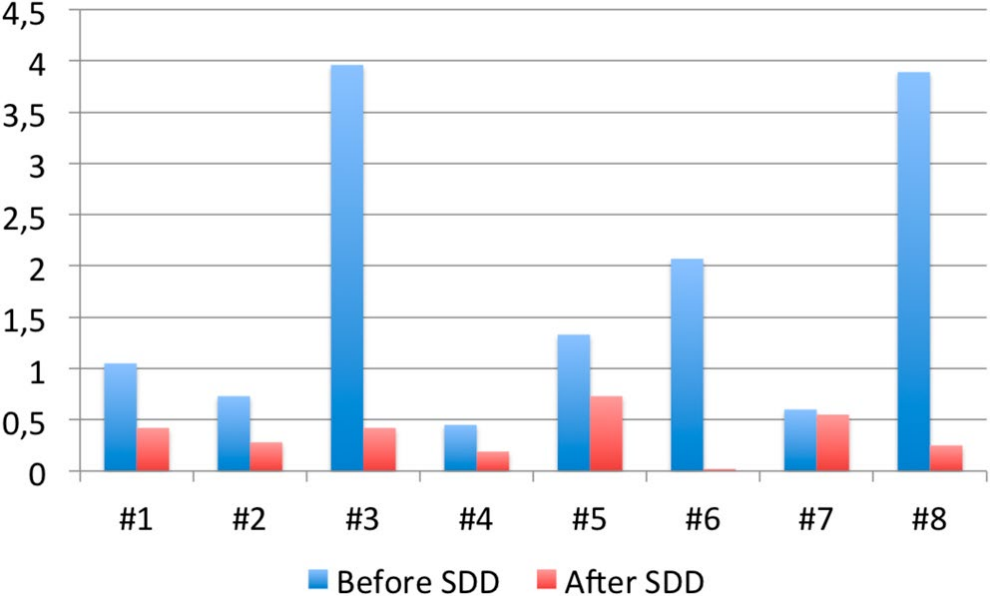
Incidence density of cholangitis (100 patients-day) before and after starting SDD in all patients (RR: 0.35;CI95%: 0.21–0.59, p: <0.001)

Period 1 included the time when patients did not receive SDD and ranged from the first documented episode of cholangitis requiring hospital admission to the start of SDD, although three patients also received oral TMP/SMZ as prophylaxis. The density or incidence rate of cholangitis requiring admission in this period ranged from 0.45 to 3.96 episodes of cholangitis per 100 patient days (Fig. 1). The incidence density of cholangitis in this period was 1.05 per 100 patient days. There were nine documented cases of bacteremia in this period, mostly due to wild-type Enterobacteriaceae. Patient #6, in one episode, presented E. coli bacteremia due to carbapenemase oxa48-producing, and Patient #7 suffered a non-bacteremic carbapenem-resistant *P. aeruginosa* infection. These multi-resistant microorganisms were not reisolated in Period 2 (after starting SDD). The overall antibiotic days received during hospitalizations for cholangitis were 13.42 days per 100 patient days.

Period 2 is the period during which patients received SDD. The overall incidence density of cholangitis requiring admission in this period was 0.37 per 100 patient days and was significantly lower than observed in Period 1 (1.05 per 100 patient days) (RR: 0.35 (95% CI: 0.21–0.59; p: <0.001, two-sided). Similarly, the duration of antibiotic therapy received during admission in this period was 3.3 per 100 patient days, which was also significantly lower than observed in Period 1 [(13.4 per 100 patient days)(RR 0.24; 95%CI: 0.20–0.30; p: <0.001, two-sided)]. The same patients who received TMP/SMZ in the first period also received it in the second period. In the second period there were ten documented bacteremias (nine in Patient #1). Patient #1 in one episode in this period developed bacteremia due to *K. pneumoniae* and *Citrobacter freundi* producing carbapenemase oxa-48; Patient #3 presented in a biliary drainage culture isolation of *Stenotrophomona maltophilia* susceptible to quinolones and TMP/SMZ. In Patient #5 *P. aeruginosa* resistant to carbapenems and susceptible to ceftolozane and Candida krusei resistant to fluconazole and E. faecium were isolated in the biliary drainage.

Follow-up days in the first period (until the initiation of SDD) ranged from 77 to 820, with a mean of 426 days. During the second period (while receiving SDD), follow-up ranged from 120 to 3070 days, with a mean of 1010 days. Follow-up was longer in the second period.

The episodes of cholangitis and antibiotherapy received by the patients in both periods included in the study were those that required admission and were well documented clinically and microbiologically. Episodes of fever, with little clinical impact, which patients controlled on an outpatient basis with oral antibiotic therapy, and which did not require hospital admission, were not included. The duration of this antibiotherapy was fewer than five days, because if the symptoms persisted after this time, hospital admission was requested. This strategy was offered to all patients after initiation of SDD and was implemented in the second period with a median of 2.3 episodes per patient. Prior to the introduction of SDD (first period) this strategy was not well established, but retrospective analysis of this period confirmed only five episodes with clinical suspicion treated on an outpatient basis in the total number of patients (median 0.6 per patient), as most episodes in this period required hospital admission.

The tolerance of SDD was very good, and in no case was its administration suspended. Only an unpleasant taste and mild initial discomfort were reported by some patients. No patient underwent major biliary surgery during follow-up, but several underwent biliary prosthesis replacements and biliary drainages were performed.

The only deceased patient was Patient #3; a liver transplant recipient with severe bile duct ischemia and external-internal biliary drainage, which he maintained until his death as a result of pulmonary neoplasia. The rest of the patients are alive and maintain a stable clinical situation apart from their episodes of cholangitis.

## Discussion

Patients suffering from recurrent cholangitis are indefinitely exposed to serious infectious episodes, with the possibility of life-threatening sepsis and septic shock. Numerous cholangiopathies are involved and several strategies have been used to prevent these processes, most with poor results. The results of this retrospective study confirm a potential benefit of SDD in reducing the number of cases and severity of cholangitis in these patients.

Given the heterogeneity of the different cholangiopathies, it is not easy to obtain reliable results from cohort studies. This study has the advantage of comparing two strategies (non-SDD vs. SDD) in the same patients and looking at the effects in the presence of the same baseline disease. In the eight patients included in this study, SDD confirmed a very significant reduction in the number of cases of cholangitis [RR: 0.35 (95% CI: 0.21–0.59)] and, therefore, in the days of intravenous antibiotherapy required for its management [RR 0.24 (95%CI: 0.20–0.30)]. The benefit was clearly related to nonabsorbable antibiotics because although three patients also received systemic antibiotic prophylaxis (TMP/SMZ) before and during SDD administration.

It is difficult to conduct clinical trials or controlled studies to analyze ways to prevent cholangitis, given the heterogeneity of conditions that favor it [1, 2, 6–8]. Among these, the clinical entity that has accumulated the most studies is the prevention of cholangitis in children with congenital biliary tract atresia undergoing Kasai portoenterostomy surgery. A recent meta-analysis, including four cohort studies and two cross-sectional studies, involved a total of 714 patients [2]; the prophylactic antibiotics used in these studies mainly included systemic antibiotics (penicillin, sulfonamide and fluoroquinolone), but also included a study with nonabsorbable oral drugs (colistin and aminoglycosides). Results showed no statistical significance; however, there was significant heterogeneity [2]. Similar results were obtained in a previous systematic review by Decharun et al. [8]. Their data suggest that patients with a Y-Roux bilio-digestive anastomosis are the most refractory in prevention strategies with absorbable or nonabsorbable antibiotics given the high bacterial inoculum present in the bilio-digestive loop. In the present series, only one patient had a biliodigestive anastomosis, but even in this patient, SDD resulted in a decrease in the incidence rate from 3.89 to 0.25 episodes per 100 days of follow-up.

Although with contradictory results, the benefit of SDD has been particularly evident in the prevention of nosocomial pneumonia in mechanically ventilated patients. However, it is noteworthy that in these studies the benefit was obtained in relation to the use of systemic antibiotics and less so to the effect of nonabsorbable antibiotics, although not in all studies [3, 4, 9, 10].

In the mid-1970s, Bodey found that the enteral administration of nonabsorbable antibiotics can eliminate the gram-negative bacilli from the gastrointestinal tract, as a result of the high drug concentrations reached in the intestinal lumen. The combination of polymyxin E (colistin) and tobramycin was chosen given its efficacy against GNB, including *Pseudomonas spp.*, and because it constituted a synergic combination in vitro [11, 12]. The administered antibiotics must meet several criteria: they must be nonabsorbable, treat flora sensitive to such antibiotics, reach bactericidal levels within the digestive tract, preserve (as far as possible) the autochthonous anaerobic flora needed to control colonization, and be safe and inexpensive [5]. The enteral antimicrobial combination of colistin, tobramycin and amphotericin B (or nystatin) complied with these criteria when it was first described [11], but the appearance of outbreaks and endemics due to MRSA and antibiotics MRGNB, in some cases, may constitute a risk.

In this series, SDD was not associated with increased selection of MRGNB. One patient had isolated *K. pneumoniae* oxa-48 bacteremia and another had carbapenem-resistant *P. aeruginosa* infection of the drainage, but SDD was maintained without selection of these bacteria in the course of the disease. Similarly, SDD helped to eradicate two severe carbapenem-resistant *P. aeruginosa* infections and one enterobacterium with oxa-48 production observed prior to SDD initiation.

The first clinical trial on the control of outbreaks with SDD was published in 1989 [13]. In this study, an endemic caused by multidrug-resistant *K. pneumoniae* was controlled using neomycin, polymyxin E, and nalidixic acid as a decontaminating formula. Ecological studies involving large sample sizes, meta-analyses and longitudinal studies with long follow-up periods have shown that the routine use of SDD is not associated with increased antibiotic resistance [3, 14, 15]. In a cluster randomized study involving 13 ICUs, information was collected over two years from 1868 patients receiving SDD and 1837 patients receiving standard care [16]. Selective digestive decontamination was associated with fewer bacteremias, and specifically bacteremias caused by multidrug-resistant flora. In the longest cohort study, evaluating the continued use of SDD over a period of 21 years in 12,053 patients, the incidence of resistant microorganisms acquired in the ICU did not increase significantly over time [15].

This study has several limitations. It is a single-center, retrospective series with a small number of patients. For a more objective analysis, only episodes of cholangitis requiring admission were included in the study, making it possible to accurately analyze microbiological isolates and the type and duration of antibiotics used. A detailed study of other possible episodes of cholangitis managed on an outpatient basis with oral antibiotics could not be performed. However, we do not believe that this could have biased the study. This strategy was used more frequently in the second period (2.3 episodes per patient) than in the first period (0.6 episodes per patient) as a consequence of the lower severity of the episodes, since if the febrile symptoms did not remit within five days the patients were invited to be hospitalized, a circumstance that occurred more frequently during the first period.

In summary, this retrospective, single-center study confirms that the use of SDD with nonabsorbable oral antibiotics (colistin, tobramycin and nystatin) is effective in the prevention of recurrent cholangitis in patients with different cholangiopathies. Its use reduces by two-third the frequency and severity of the processes, and by three-quarter the total intravenous antibiotic duration required to treat these episodes. Therefore, treatment can be completed in a first step with absorbable oral antibiotics (beta-lactams, quinolones, etc.) for the management of the less severe and more frequent forms that patients have under this prophylaxis. It is possible that their efficacy may be lower in certain cholangiopathies with high bacterial inoculum such as those with Y-Roux bilio-digestive anastomosis. In addition to being effective, its application is patient-friendly and microbiologically safe, because it is not associated with an increased risk of selection of multi-resistant agents.

## Data Availability

No datasets were generated or analysed during the current study.

## Abbreviations

SDD: Selective decontamination of the digestive tract
TMP/SMZ: Trimethoprim/sulfamethoxazole
FOLFOX: Folinic acid, fluorouracil, and oxaliplatin
Pat: Patient
CCT: Cholecystectomy
pds: Patient days
AB: Antibiotics
Tx: Transplant
